# Recent therapeutic advances in chronic lymphocytic leukemia

**DOI:** 10.12688/f1000research.11618.1

**Published:** 2017-10-31

**Authors:** Prithviraj Bose, Varsha Gandhi

**Affiliations:** 1 Department of Leukemia, The University of Texas MD Anderson Cancer Center, Houston, TX, USA; 2 Department of Experimental Therapeutics, The University of Texas MD Anderson Cancer Center, Houston, TX, USA

**Keywords:** CLL, BCR pathway, ibrutinib, BTK, Bcl-2, venetoclax, CD20 antibody, PI3K

## Abstract

The last several years have witnessed a paradigm shift in the management of patients with chronic lymphocytic leukemia (CLL). The course of this very heterogeneous disease, traditionally treated with chemotherapeutic agents usually in combination with rituximab, typically has been characterized by remissions and relapses, and survival times vary greatly, depending on intrinsic biological attributes of the leukemia. The developments of the last few years have been transformative, ushering in an era of novel, molecularly targeted therapies, made possible by extensive efforts to elucidate the biology of the disease that predated the new targeted drugs. Thus, successful therapeutic targeting of the B-cell receptor signaling pathway and of the Bcl-2 anti-apoptotic protein with small molecules has now made chemotherapy-free approaches possible, hopefully mitigating the risk of development of therapy-related myeloid neoplasms and making eventual cure of CLL with the use of optimal drug combinations a realistic goal. Most importantly, these therapies have demonstrated unprecedented efficacy in patients with deletion 17p/TP53 mutation, a subset that historically has been very difficult to treat. However, as we gain more experience with the newer agents, unique safety concerns and resistance mechanisms have emerged, as has the issue of cost, as these expensive drugs are currently administered indefinitely. Accordingly, novel laboratory-based strategies and clinical trial designs are being explored to address these issues. The availability of whole exome/genome sequencing has given us profound insights into the mutational landscape of CLL. In this article, we highlight some of the most impactful advances since this topic was last reviewed in this journal.

## Introduction

The pace of discovery, with respect to both biology and therapeutic targets, as well as that of drug approval, in chronic lymphocytic leukemia (CLL) has been particularly rapid in recent years, so much so that therapeutic advances made in the treatment of CLL were named the “advance of the year” in 2015 by the American Society of Clinical Oncology. This topic was last reviewed in this journal in 2014 by Stilgenbauer and colleagues ^1^. In this article, we review more recent developments, focusing on new genomic information, novel targeted therapies, and emerging targets and drugs/drug combinations as well as new information that has accumulated on agents that had just been approved or whose approval was imminent when the last review was written, namely ibrutinib and idelalisib.

## Recent insights into the chronic lymphocytic leukemia genome and integration of mutational information into risk stratification systems

A lot has been learned on the topic of somatic mutations in CLL since the initial reports in 2011 (reviewed in 2). Two recent studies employing next-generation sequencing (either whole exome or whole genome) molecularly annotated nearly 1,000 CLL samples, identifying previously unrecognized putative driver mutations (for example, in *ZNF292*, *ZMYM3*, *ARID1A*, *PTPN11*, *RPS15*, and *IKZF3*), including some in non-coding DNA (for example, the 3′ region of *NOTCH1* that leads to aberrant splicing and increased NOTCH1 activity, and an enhancer located on chromosome 9p13 that results in reduced expression of PAX5), and many subclonal mutations and documenting frequent clonal evolution, even in the absence of therapy ^3, 4^. *SF3B1* and *NOTCH1* (previously described) represented the most frequently mutated genes in these studies ^3, 4^. The functional consequences of *SF3B1* mutations, which have been associated with faster disease progression and poor overall survival (OS) in CLL ^5^, and their near mutual exclusivity with *NOTCH1* mutations ^6^ are also now better understood. The former, often associated with del(11q) ^7^, lead to alternative splicing ^8, 9^, impairment of the DNA damage response network ^10^, and dysregulation of NOTCH signaling and telomere biology ^11^. In the German CLL8 trial that compared fludarabine, cyclophosphamide, and rituximab (FCR) with fludarabine and cyclophosphamide (FC) and showed a survival advantage for the chemoimmunotherapy (CIT) combination ^12^, *NOTCH1* mutations were associated with a lower rate of response to rituximab and the lack of a survival benefit from the addition of rituximab ^6^. *NOTCH1* mutations, which are most frequently present in CLL patients with trisomy 12 ^13, 14^, have subsequently been shown to lead to epigenetic dysregulation, resulting in lower CD20 expression ^15^. Del(13q), del(11q), trisomy 12, and mutations in the gene encoding MYD88, an adaptor protein in the Toll-like receptor pathway, appear to represent early genomic lesions with potential roles in CLL initiation, whereas mutations in *SF3B1*, the second allele of *ATM* and *TP53* are likely to be later genetic events ^4, 16^.

*TP53* mutations correlate strongly with del(17p) ^6^, just as *ATM* mutations do with del(11q) ^17^. Though relatively infrequent in treatment-naïve CLL, *TP53* mutations and deletions are significantly enriched for after CIT ^18^. *TP53* mutations are independently associated with worse progression-free survival (PFS) and OS in the setting of first-line CIT ^6^ and have been incorporated into the recently published CLL-International Prognostic Index (CLL-IPI) ^19^. This five-factor prognostic scoring system takes into account *TP53* status (no abnormalities versus del(17p) or *TP53* mutation or both), the mutational status of the immunoglobulin heavy chain variable region ( *IGHV*), age, clinical stage, and serum beta-2-microglobulin and discriminates between four risk groups with 5-year survival rates ranging from 23.3% to 93.2% ^19^. A simple and user-friendly “biomarkers-only” prognostic model using only *IGHV* mutational status and cytogenetics by interphase fluorescence *in situ* hybridization was recently reported to perform as well as the CLL-IPI: in 524 unselected subjects with CLL, 10-year OS rates were 82% in the low-risk group, 52% in the intermediate-risk group, and 27% in the high-risk group; the model was validated in two independent cohorts, one of which was composed only of patients with Binet stage A CLL ^20^. Efforts have also been made to integrate mutational and cytogenetic information into a genetic prognostic model for patients with CLL. This model, which remained valid at any time from diagnosis, delineated four risk groups with very different 10-year survival probabilities (29%–69.3%): high risk, comprising patients with *TP53* or *BIRC3* abnormalities or both; intermediate risk, characterized by *NOTCH1* or *SF3B1* mutations or del(11q22-23) or a combination of these; low risk, consisting of patients with trisomy 12 or normal cytogenetics; and very low risk, patients with isolated del(13q14) ^21^. Among patients with early-stage disease, high CLL-cell birth rates are associated with shorter treatment-free survival ^22^.

## Recent advances in targeting cell surface proteins

The remarkable clinical benefits of the addition of rituximab (anti-CD20 monoclonal antibody) to chemotherapy ^12, 23^ generated tremendous interest in this class of agents. Originally approved for the treatment of CLL refractory to both fludarabine and alemtuzumab, ofatumumab (Arzerra ^®^), a fully human anti-CD20 monoclonal antibody that binds to a different epitope than rituximab, has recently been approved, along with chlorambucil, for the first-line treatment of CLL in patients deemed unfit for fludarabine-based therapy, as monotherapy for the extended treatment (“maintenance”) of patients with recurrent or progressive CLL who have received at least two lines of therapy and achieved a complete response (CR) or partial response (PR) to their most recent therapy, and in combination with FC for the treatment of patients with relapsed CLL, on the basis of the COMPLEMENT-1 ^24^, PROLONG ^25^, and COMPLEMENT-2 ^26^ trials, respectively. However, because this agent has never been shown to be superior to rituximab in a head-to-head comparison, although such data exist for the type II glycoengineered anti-CD20 monoclonal antibody obinutuzumab (Gazyva™) ^27^, and because of the advent of the new small-molecule targeted therapies, enthusiasm for the use of ofatumumab in CLL has waned considerably. Like obinutuzumab, ublituximab (formerly TG-1101) is an anti-CD20 monoclonal antibody with enhanced antibody-dependent cellular cytotoxicity (ADCC); the lower fucose content of its Fc domain improves its binding to FcγRIIIA ^28, 29^. In a phase 1/2 trial in patients with rituximab-relapsed or -refractory B-cell non-Hodgkin’s lymphoma (NHL) (n = 27) or CLL (n = 8), the most common adverse events (AEs) were infusion-related reactions (40%), fatigue (37%), pyrexia (29%), diarrhea (26%), neutropenia (14%), and anemia (11%) ^30^. In a phase 2 study in relapsed or refractory (R/R) CLL (n = 45) in which patients received six cycles of ublituximab in conjunction with ibrutinib, the overall response rate (ORR) was 88% at 6 months ^31^. Among 20 patients with del(17p), del(11q), or *TP53* mutation, the ORR was 95%, and three of these patients attained minimal residual disease (MRD) negativity. This combination is now being compared with ibrutinib alone in the GENUINE phase 3 study (NCT02301156) in patients with R/R CLL bearing the above genetically high-risk features. At a median follow-up of 12 months, the best ORR by independent central review was 80% for ublituximab plus ibrutinib compared with 47% for ibrutinib alone ^32^.

Otlertuzumab is a humanized, anti-CD37 monospecific protein therapeutic that has been studied in a phase 1 trial in 83 (mostly previously treated) patients with CLL ^33^. Twelve (20%) of sixty-one evaluable patients responded; all responses were partial and were more common in less heavily pretreated patients. This agent, in combination with bendamustine, has been compared with bendamustine alone in a small (n = 65) randomized phase 2 study in relapsed CLL ^34^. ORRs were 69% in the combination arm and 39% for bendamustine alone ( *P* = 0.025), which also translated into a PFS benefit (median of 15.9 versus 10.2 months, *P* = 0.0192) for the combination. An ongoing phase 1b trial (NCT01644253) with many cohorts is studying this agent in combination with rituximab, obinutuzumab, ibrutinib, and idelalisib plus rituximab.

## Targeting Bruton’s tyrosine kinase: ibrutinib and beyond

The prototypical irreversible Bruton’s tyrosine kinase (BTK) inhibitor ibrutinib is currently approved for all patients with CLL on the basis of the results of the RESONATE ^35^, RESONATE-2 ^36^, and RESONATE-17 ^37^ trials. RESONATE compared ibrutinib with ofatumumab in 391 patients with R/R CLL or small lymphocytic lymphoma (SLL) and showed large improvements in ORRs and both PFS and OS ^35^, as did RESONATE-2, which compared ibrutinib with chlorambucil in 269 treatment-naïve, older (at least 65 years of age) patients with CLL or SLL ^36^. RESONATE-17 was a single-arm study of ibrutinib in 144 patients with R/R CLL or SLL and del(17p) ^37^. The ORR by independent review at the prespecified primary analysis after a median follow-up of 11.5 months was 64% (83% by investigator assessment) ^37^. After a median follow-up of 27.6 months (n = 120), the investigator-assessed ORR was 83%, 24-month PFS was 63%, and 24-month OS was 75% ^37^. Of note, complex karyotype may be a stronger predictor of inferior outcomes than del(17p) among patients with R/R CLL in the setting of ibrutinib therapy ^38^; however, not all studies have arrived at similar conclusions ^39^. Overall, responses to ibrutinib are durable and improve over time, while toxicities such as treatment-emergent grade 3/4 cytopenias, fatigue, and infections diminish ^40^. Disease progression is uncommon up to three years (median) of follow-up and mainly occurs in patients with relapsed disease harboring del(17p) or del(11q) ^40^.

While ibrutinib’s mobilizing effects on CLL cells resident in protective nodal microenvironmental niches and interference with their homing to the same are well known ^41^, an elegant study using deuterated water labeling has shown that the drug also has profound and immediate anti-proliferative and apoptosis-inducing actions on CLL cells ^42^. Ibrutinib is more efficient at clearing lymph node than blood or marrow disease, and recent work has demonstrated that the highest rate of CLL cell proliferation occurs in the lymph nodes ^43^. However, the “redistribution lymphocytosis” caused by ibrutinib, which has led to the introduction of the CLL response category “partial response with lymphocytosis (PR _L_)” ^44^, does not have any adverse long-term consequences even if prolonged, and these cells eventually die from the lack of microenvironmental pro-survival signals ^45^.

### Ibrutinib resistance and therapeutic options

Resistance to ibrutinib has been attributed to acquired mutations in BTK that only allow the kinase to be reversibly bound by ibrutinib, as well as downstream mutations in phospholipase C gamma 2 (PLCγ2) that reactivate B-cell receptor (BCR) signaling despite inhibition of BTK function by ibrutinib ^46– 48^. More recent work has identified clonal evolution, particularly the emergence of del(8p) clones harboring additional driver mutations (in *EP300*, *MLL2*, and *EIF2A*), as an additional mechanism of development of resistance to ibrutinib ^49^. Outcomes after ibrutinib discontinuation have been reported to be poor ^50, 51^, particularly for patients discontinuing because of progression and especially Richter’s transformation (RT) of their CLL, but appear to be improving with the availability of newer and effective salvage options ^52^. RTs usually occur early, whereas CLL progressions generally tend to be later events ^51^. Clinical data are available supporting the efficacy of commercially available drugs such as idelalisib and venetoclax in the setting of ibrutinib resistance or intolerance (discussed below) ^53, 54^ as well as investigational agents such as duvelisib (formerly IPI-145, discussed below) ^55^ and entospletinib (formerly GS-9973, a spleen tyrosine kinase inhibitor) ^56^.

Additionally, protein kinase C beta inhibitors ^57^, heat shock protein 90 inhibitors ^58^, and selective inhibitors of nuclear export ^59^ hold promise in preclinical studies to overcome ibrutinib resistance in CLL. Finally, a number of reversible BTK inhibitors that bind outside of the C481 residue targeted by ibrutinib and therefore are able to effectively inhibit the resistant C481S mutant are in development ^60– 62^.

### Other Bruton’s tyrosine kinase inhibitors

Ibrutinib inhibits a number of kinases besides BTK, its primary therapeutic target in CLL ^63^, including some at subnanomolar concentrations ^64^. Some of these off-target effects of ibrutinib may be responsible for some of its unique toxicities (for example, atrial fibrillation ^65^ and bleeding ^66– 68^). Thus, there is considerable interest in developing more selective inhibitors of BTK. Among these, acalabrutinib (formerly ACP-196) is farthest along in clinical development (phase 3). Consistent with the concept that acalabrutinib is a more selective BTK inhibitor, preclinical studies have demonstrated that ibrutinib and acalabrutinib have similar biological activity in primary CLL cells but appear to have differences in their impact on normal T cells ^69^. In a phase 1–2 study in 61 patients with relapsed CLL and a median of three prior therapies, acalabrutinib produced an ORR of 95% (85% PR and 10% PR _L_); all patients with del(17p) responded ^70^. In a phase 1 trial of another selective BTK inhibitor, ONO/GS-4059, in 90 patients with R/R B-cell malignancies, 24 (96%) of 25 evaluable patients with CLL responded, and the median duration of response was 80 weeks ^71^. Yet another such agent is BGB-3111 ^72^. The ORR to this agent among 29 evaluable patients with R/R CLL/SLL was 90% (79% PR and 10% PR _L_) ^73^. With the short follow-up reported thus far, all three agents have been very well tolerated; however, there have been cases of major bleeding and atrial fibrillation reported with ONO/GS-4059 and BGB-3111 ^71, 73^.

### Ibrutinib dose and biological activity

Another approach to minimize the off-target toxicities of ibrutinib while preserving efficacy may be the exploration of lower doses ^74^. This is based on the observations that ibrutinib doses of at least 2.5 mg/kg per day were sufficient to achieve at least 95% BTK occupancy in the phase 1 trial in patients with R/R B-cell malignancies ^75^ and that BTK levels decline over time in CLL cells from ibrutinib-treated patients ^76, 77^. Since ibrutinib is an irreversible inhibitor of BTK and binds to BTK in a 1:1 stoichiometric ratio, this would imply the need for lower doses of ibrutinib over time as BTK levels decline. Therefore, continued dosing at 420 mg/day could lead to greater off-target binding and toxicity ^74^. Our group is currently studying the pharmacodynamic correlates, including BTK occupancy, of progressively lower dosing of ibrutinib in patients with CLL in the context of a pilot study (NCT02801578). Importantly, a retrospective multi-institutional “real world” study published recently showed that reduced dose ibrutinib—defined as sustained (for at least 2 months) dosing at less than 420 mg/day, either at treatment initiation or within 3 months from starting ibrutinib—did not compromise outcomes (that is, ORR or PFS) ^78^. Toxicity or physician preference drove the decision to reduce the dose of ibrutinib in the vast majority of cases. In contrast, missing ** at least 8 consecutive days of ibrutinib has been correlated with shorter median PFS ^79^.

### Combination strategies involving ibrutinib

Ibrutinib has been combined with anti-CD20 monoclonal antibodies in non-comparative clinical trials ^80, 81^, and although early fears of antagonism of rituximab-mediated ADCC by ibrutinib based on preclinical studies ^82^ have been laid to rest, the incremental benefit of this approach (versus ibrutinib alone) is not clear. Similarly, the HELIOS study in 578 patients with R/R non-del(17p) CLL or SLL showed improved PFS with the addition of ibrutinib to bendamustine and rituximab (BR), but this regimen does not appear to offer meaningful advantages over ibrutinib monotherapy, except when a rapid response is clinically desirable ^83^. One US cooperative group study is investigating BR, ibrutinib alone, or ibrutinib plus rituximab in previously untreated patients at least 65 years of age (NCT01886872), while another is comparing frontline FCR with ibrutinib plus rituximab in patients 18 to 70 years of age (NCT02048813). Accrual to both of these studies is complete and results are awaited.

## Targeting phosphatidylinositol-3-kinase: idelalisib and newer inhibitors

### Idelalisib

The first-in-class phosphatidylinositol-3-kinase (PI3K) delta isoform-specific inhibitor idelalisib (Zydelig ^®^) was approved in conjunction with rituximab by the US Food and Drug Administration (FDA) in 2014 for patients with relapsed CLL for whom rituximab alone would be considered appropriate therapy (because of co-morbidities) on the basis of the results of a pivotal phase 3 study in which the combination significantly improved both PFS and OS over rituximab plus placebo, so much so that the trial was stopped early ^84^. Like ibrutinib, idelalisib blocks signaling through the BCR pathway and is efficacious in patients harboring del(17p) or *TP53* aberrations. We have recently shown that in mantle cell lymphoma cell lines and patient-derived samples, idelalisib inhibits protein synthesis, which correlates with reductions in AKT (the immediate downstream effector of PI3K) and mitogen-activated protein kinase kinase (MEK) phosphorylation ^85^. Idelalisib synergizes with bendamustine in primary CLL cells, increasing DNA damage and suppressing transcription of the anti-apoptotic protein myeloid cell leukemia 1 (MCL-1) ^86^. Indeed, in a recently reported phase 3 trial (n = 416), the combination of idelalisib with BR markedly enhanced PFS in patients with R/R CLL (median of 20.8 versus 11.1 months for BR plus placebo after a median follow-up of 14 months) ^87^. Idelalisib plus ofatumumab has also been compared with ofatumumab alone in 261 patients with R/R CLL (median number of prior therapies = 3) in a randomized phase 3 trial ^88^. The primary analysis of this trial showed a doubling of median PFS (16.3 versus 8 months) in the combination arm.

The use of idelalisib in patients with CLL has been constrained by toxicity concerns, particularly given the superior safety profile of ibrutinib. The US prescribing information for idelalisib contains a black-box warning for fatal or serious hepatotoxicity or both (11%–18%), diarrhea/colitis (14%–19%), pneumonitis (4%), infections (21%–36%), and intestinal perforation, and a high degree of vigilance for these AEs is essential in patients receiving idelalisib. The incidence of hepatotoxicity, believed to be immune-mediated, has been reported to be particularly high in the setting of frontline idelalisib monotherapy; 19 (79%) of 24 subjects experienced some degree of transaminitis and 13 (54%) had at least grade 3 transaminitis in a recent study ^89^. The median time to development of transaminitis was 28 days, and younger age and mutated *IGHV* status predicted for this complication. Very recently, it was reported that PI3K delta blockade by either idelalisib or duvelisib (discussed below), through upregulation of the B cell–specific enzyme activation-induced cytidine deaminase, induces genomic instability in normal and neoplastic B cells, which could lead to lymphomagenesis given the potential for patients to be on these drugs for prolonged periods ^90^.

### Other PI3K inhibitors

TGR-1202 is a novel PI3K delta inhibitor with an improved safety profile, particularly with regard to hepatotoxicity and colitis. In a phase 1 study in patients with R/R CLL or NHL, 10 (63%) of 16 evaluable patients with CLL achieved a PR. This agent has been combined with ublituximab: of 10 R/R CLL patients who received the combination, all were progression-free at a median of 8 months when these data were last presented. A pivotal phase 3 trial (UNITY-CLL) comparing ublituximab and TGR-1202 with chlorambucil and obinutuzumab is under way (NCT02612311), as are a number of other studies combining TGR-1202 or TGR-1202 plus ublituximab with ibrutinib, bendamustine, or pembrolizumab.

Duvelisib (reviewed in 91) is a small-molecule inhibitor of both the delta and gamma isoforms of PI3K that potently and selectively inhibits the proliferation of primary CLL cells, induces apoptosis, and interferes with the homing capabilities of CLL cells through blockade of BCR signaling ^92^. Preclinically, duvelisib impairs the viability of T cells and natural killer cells and decreases the production by activated T cells of inflammatory and anti-apoptotic cytokines ^93^. ORRs to single-agent duvelisib have ranged from 55% in heavily pretreated patients with R/R CLL to 82% in previously untreated patients; as with ibrutinib and idelalisib, the vast majority of responses have been partial ^94, 95^. Duvelisib has been studied in combination with bendamustine, rituximab, and BR in patients with R/R CLL or indolent NHL with good tolerability ^96^. In treatment-naïve patients, the addition of duvelisib to FCR appears to substantially increase the rate of MRD negativity ^97^, a strong surrogate for long-term outcome in CLL ^98^. Results of the completed phase 3 DUO study (NCT02004522) comparing duvelisib with ofatumumab in 319 patients with R/R CLL or SLL are expected soon; the trial has met its primary endpoint of significantly improved PFS in the duvelisib arm (13.3 versus 9.9 months), according to topline results released recently by the company.

## Targeting B-cell lymphoma 2 with venetoclax

CLL cells are exquisitely dependent on the B-cell lymphoma 2 (BCL-2) anti-apoptotic protein for survival ^99^, and the “BH3-mimetic” navitoclax, an antagonist of both BCL-2 and BCL-xL, showed promising activity in patients with R/R CLL ^100^, but the development of this agent was hampered by the occurrence of dose-limiting thrombocytopenia in all clinical trials, an on-target consequence of the drug’s action on platelets and megakaryocytes, which rely on BCL-xL for survival ^101^. These observations led to the development of venetoclax (Venclexta™, formerly ABT-199), a highly BCL-2–selective antagonist that spares platelets ^102^, by reverse engineering of navitoclax.

In a phase 1 trial with dose escalation and expansion phases, 116 patients with R/R CLL or SLL (but none previously treated with BCR inhibitors) received venetoclax ^103^. The ORR was 79%, including CRs in 20% of patients, a quarter of which were MRD-negative. Clinical tumor lysis syndrome (TLS), fatal in one case, occurred in three of 56 patients in the dose escalation phase, but did not recur in the dose expansion phase (n = 60), after a careful dose ramp-up to a maximum of 400 mg daily was instituted. Mild diarrhea (52%), upper respiratory infection (48%), nausea (47%), and grade 3/4 neutropenia (41%) were frequent. Venetoclax was approved in 2016 by the FDA for patients with CLL with del(17p) based on an ORR of 79.4% in a separate study (n = 107) carried out exclusively in patients with R/R del(17p) CLL ^104^. The CR rate, with or without complete count recovery, in this study was only 8%, and only 5% of the patients had received prior BCR inhibitors. Grade 3/4 neutropenia was very common (40%), and grade 3/4 infections, anemia, and thrombocytopenia occurred in 20%, 18%, and 15% of patients, respectively. According to the current label, the dose of venetoclax should be ramped up over a 4- to 5-week period from 20 to 400 mg daily to minimize the risk of TLS. Venetoclax was combined with rituximab in a phase 1 trial in 49 patients with R/R CLL or SLL, and the recommended phase 2 dose (RP2D) was found to be 400 mg/day in this setting as well ^105^. Clinical TLS occurred in two patients who started the dose ramp-up at 50 mg/day, and resulted in one death. The ORR was 86% and MRD negativity was attained in 57%. Twenty-five patients (51%) achieved CR and 20 of them (80%) were MRD-negative. Grade 1/2 upper respiratory infection, diarrhea, and nausea were very frequent, affecting 57%, 55%, and 51% of patients, respectively. Grade 3/4 neutropenia affected 53% of patients, and febrile neutropenia 12%; 14% and 16% of patients, respectively, had grade 3/4 anemia and thrombocytopenia.

An important question not addressed by the above studies concerns the efficacy of venetoclax in patients failing BCR inhibitors. This is the subject of an ongoing study, preliminary results of which have been presented ^53^. The study enrolled only patients who had relapsed or were refractory to ibrutinib (n = 43) or idelalisib (n = 21). The ORRs to venetoclax monotherapy were 70% in the prior ibrutinib group and 48% in the prior idelalisib group. Only one CR (with incomplete count recovery) was documented by independent review—in the prior ibrutinib group.

## Combination strategies and the road to a cure in chronic lymphocytic leukemia

Long-term (more than 10 years) relapse-free remissions are already achievable with FCR in the subset of patients with favorable genomic prognostic factors (that is, mutated *IGHV*, del(13q), normal cytogenetics, or trisomy 12) ^106^. Ongoing clinical trials are attempting to further improve outcomes by adding ibrutinib to frontline FCR, followed by ibrutinib maintenance in younger, fit patients ^107^, or adding ibrutinib and replacing rituximab with the more potent obinutuzumab, while reducing the exposure to cytotoxic chemotherapy (to mitigate the real risk of therapy-related myeloid neoplasms) ^108^, as well as limiting the duration of ibrutinib maintenance using an MRD-driven approach in previously untreated patients with mutated *IGHV* and without del(17p) ^109^. Achievement of MRD negativity has become widely established as a necessary first step to an eventual cure of CLL (reviewed in 110).

Preclinical studies from our group ^111^ and others ^112^ have demonstrated synergism between ibrutinib and venetoclax in CLL. The clinical efficacy profiles of these two oral agents also complement each other well, as ibrutinib is particularly effective at clearing nodal disease and less so at clearing marrow disease, whereas venetoclax has the opposite profile and also does not cause the redistribution lymphocytosis typical of BCR inhibitors. Furthermore, CR rates with ibrutinib monotherapy, at least in the R/R setting, are very low ^35, 113^ and clearly better with venetoclax, which is additionally capable of inducing MRD negativity on its own ^103^. These observations make this a particularly attractive combination, which is being studied in several ongoing clinical trials in both the frontline and R/R settings (NCT02756897, NCT03045328, and NCT02910583). Other trials are studying the triple combination of ibrutinib, venetoclax, and obinutuzumab (NCT02758665 and NCT02427451). Early results demonstrate tolerability of the triple combination, and the RP2D of venetoclax is the same as the approved monotherapy dose (that is, 400 mg/day) ^114^. Aside from toxicity concerns, the enormous economic burden of indefinite therapy of CLL with the new oral targeted agents ^115^ makes achievement of a deep response with the use of an optimal combination regimen for a finite duration with, hopefully, a durable treatment-free remission an important goal.

Duvelisib has also been shown to synergize with venetoclax in induction of apoptosis of CLL cells ^116^. However, there are no ongoing clinical studies of PI3K delta inhibitors in combination with venetoclax; one involving duvelisib was withdrawn. This could reflect the substantial toxicity concerns with idelalisib discussed above, as well as the recent findings of induction of genomic instability in B cells by both idelalisib and duvelisib ^90^. If indeed TGR-1202 turns out to have a much improved safety profile in the clinic, it is possible that the combination of this agent with venetoclax will be pursued in trials.

## Emerging drug targets

Single-agent immune checkpoint blockade with pembrolizumab (Keytruda ^®^), a monoclonal antibody directed against programmed death 1 (PD-1), was recently shown to have substantial clinical activity in patients with RT, a difficult-to-treat, poor-prognosis entity, but not in CLL ^117^. As noted above, this agent is also being studied in combination with ublituximab plus TGR-1202 in patients with R/R CLL or RT (NCT02535286). Additionally, it is being evaluated in combination with ibrutinib or idelalisib in patients with R/R CLL (NCT02332980). The anti–PD-1 monoclonal antibody nivolumab (Opdivo ^®^) is also being evaluated in combination with ibrutinib in both patients with RT and R/R or high-risk CLL (NCT02420912). Studies in mouse models of lymphoma support the combination of ibrutinib with immune checkpoint blockade ^118^. This is based, in part, on the inhibition by ibrutinib of interleukin-2-inducible kinase (ITK) in T cells, which skews T-cell immune responses away from Th2 and toward a Th1 phenotype ^119^.

Resistance to venetoclax is largely driven by MCL-1 (reviewed in 120); for years, this anti-apoptotic protein has eluded therapeutic targeting. However, a number of clinical candidate compounds capable of directly antagonizing the function of MCL-1 are now on the horizon ^121– 123^ and hopefully will be available in the future for combination with venetoclax. Furthermore, therapy with BCR axis inhibitors such as ibrutinib ^111^, acalabrutinib ^69^, idelalisib ^124^, and duvelisib ^116^ results in a decline in MCL-1 protein levels in CLL cells, providing a mechanism-based rationale to combine them with venetoclax. Another strategy involves downregulating this short-lived anti-apoptotic protein through transcriptional repression, achievable by inhibition of cyclin-dependent kinase 9 (CDK9) ^125^. While “pan”-CDK inhibitors have displayed clear evidence of activity in CLL ^126, 127^, current efforts in this area are focusing on developing agents that are selective for CDK9. CYC065, for example, is highly selective for CDK2 and CDK9.

A relatively new therapeutic target in CLL is colony-stimulating factor 1R (CSF1R), expressed on tumor-associated macrophages (TAMs), and macrophage killing by CSF1R blockade induces CLL cell death, primarily through the tumor necrosis factor pathway ^128^. TAMs provide support to CLL cells via a PI3K-AKT-mammalian target of rapamycin (mTOR)-dependent translational upregulation of MCL-1 ^129^. Both small-molecule kinase inhibitors (for example, pexidartinib ^130^ and BLZ945 ^131^) and monoclonal antibodies targeting CSF1R are in development for various tumor types.

## Conclusions

The past few years have seen enormous advancements in our understanding of CLL biology and drug discovery and clinical development. The advent of the BCR inhibitors and venetoclax has fundamentally changed the paradigm of CLL management and brought unprecedented benefits to patients, particularly those with historically poor outcomes with CIT (for example, those with del(17p) or *TP53* abnormalities). The challenges facing the field in the coming years will be how to optimally combine and sequence these and newer agents so as to achieve high rates of MRD eradication, hopefully enabling treatment discontinuation and translating to long-term relapse-free survival. Identification of mechanisms of resistance to the novel targeted agents and their abrogation, along with effective treatment and prevention of RT, will likely become a major focus of CLL research in the years to come. New drugs targeted against CD37, MCL-1, CDK9, CSF1R, and so on hold promise for an even more robust therapeutic armamentarium in the near future.
